# Cryo‐EM targets in CASP14

**DOI:** 10.1002/prot.26216

**Published:** 2021-09-16

**Authors:** Tristan Cragnolini, Andriy Kryshtafovych, Maya Topf

**Affiliations:** ^1^ Institute of Structural and Molecular Biology Birkbeck, University College London London UK; ^2^ Genome Center University of California, Davis Davis California USA; ^3^ Center for Structural Systems Biology Leibniz‐Institut für Experimentelle Virologie and Universitätsklinikum Hamburg‐Eppendorf (UKE) Hamburg Germany

**Keywords:** CASP, cryo‐EM, electron microscopy, model evaluation, protein structure prediction

## Abstract

Structures of seven CASP14 targets were determined using cryo‐electron microscopy (cryo‐EM) technique with resolution between 2.1 and 3.8 Å. We provide an evaluation of the submitted models versus the experimental data (cryo‐EM density maps) and experimental reference structures built into the maps. The accuracy of models is measured in terms of coordinate‐to‐density and coordinate‐to‐coordinate fit. A‐posteriori refinement of the most accurate models in their corresponding cryo‐EM density resulted in structures that are close to the reference structure, including some regions with better fit to the density. Regions that were found to be less “refineable” correlate well with regions of high diversity between the CASP models and low goodness‐of‐fit to density in the reference structure.

## 1 INTRODUCTION

The community‐wide experiment on the Critical Assessment of techniques for protein Structure Prediction (CASP) provides an independent mechanism for assessing methods in protein structure prediction. The experiment is an unbiased testing ground with the credibility of results ensured through the “blind prediction” mechanism requesting that all predictions are made before structures become known to the public. To get a supply of modeling targets, CASP relies on the help of the experimental structural biology community. Since CASP started in 1994, the community has provided more than 1100 sequences of soon‐to‐be‐solved protein structures as prediction targets, including 84 sequences offered for the latest, 14th round of CASP. Historically, the vast majority of targets were coming from the crystallography structure determination groups. With the recent advances in cryo‐EM structure determination, the number of structures solved using this technique is growing rapidly, nearly doubling annually and approaching 8000 entries in the Protein Data Bank as of June 2021 (https://www.rcsb.org/stats/growth/growth-em).

In response to this growth, CASP expanded its target supplier network to engage more structural biologists from the cryo‐EM community. As a result, a sizable share of CASP targets now comes from the cryo‐EM field. In CASP13, 8% of targets were determined by cryo‐EM,^1^ while in CASP14—13% (yielding 22% of evaluation units). Percent‐wise, this is more than the share of cryo‐EM structures in the whole PDB (currently 4%) although this share is rising, with nearly 20% of structures submitted in 2021 coming from cryo‐EM.^2^ An adequate representation of cryo‐EM structures in CASP is important for several reasons. First, cryo‐EM targets differ from other CASP targets in terms of their size and complexity of architecture, and therefore their unproportional share may introduce bias in the evaluation. Second, reference structures from cryo‐EM studies often have higher coordinate uncertainty due to lower or nonuniform resolution and as such, may represent multiple conformation in one target. For these reasons, CASP organizers thought it useful to conduct a separate evaluation of cryo‐EM targets with the emphasis on model fit to the experimental data per se and not the coordinates derived from these data (reference structure).

Here, we assess the fit of the submitted models to the cryo‐EM density maps, and compare the best‐fit models (with and without refinement in the density) to the corresponding reference structures provided by the experimentalists. We also compare the performance of CASP14 tertiary structure prediction methods on all targets versus the cryo‐EM targets in the traditional CASP way (vs the reference structure) to assure no abnormalities due to specifics of the structure determination approach.

## 2 MATERIALS AND METHODS

### 2.1 Participants and predictions

Modeling of cryo‐EM targets was a part of the general CASP14 modeling experiment. Models of cryo‐EM targets were generated the same way as models of other CASP targets, that is, based solely on sequence. 142 groups submitted 513 models on cryo‐EM targets representing multimeric complexes, and 3576 models on their subunits.

### 2.2 Cryo‐EM targets in CASP14

**FIGURE 1:**
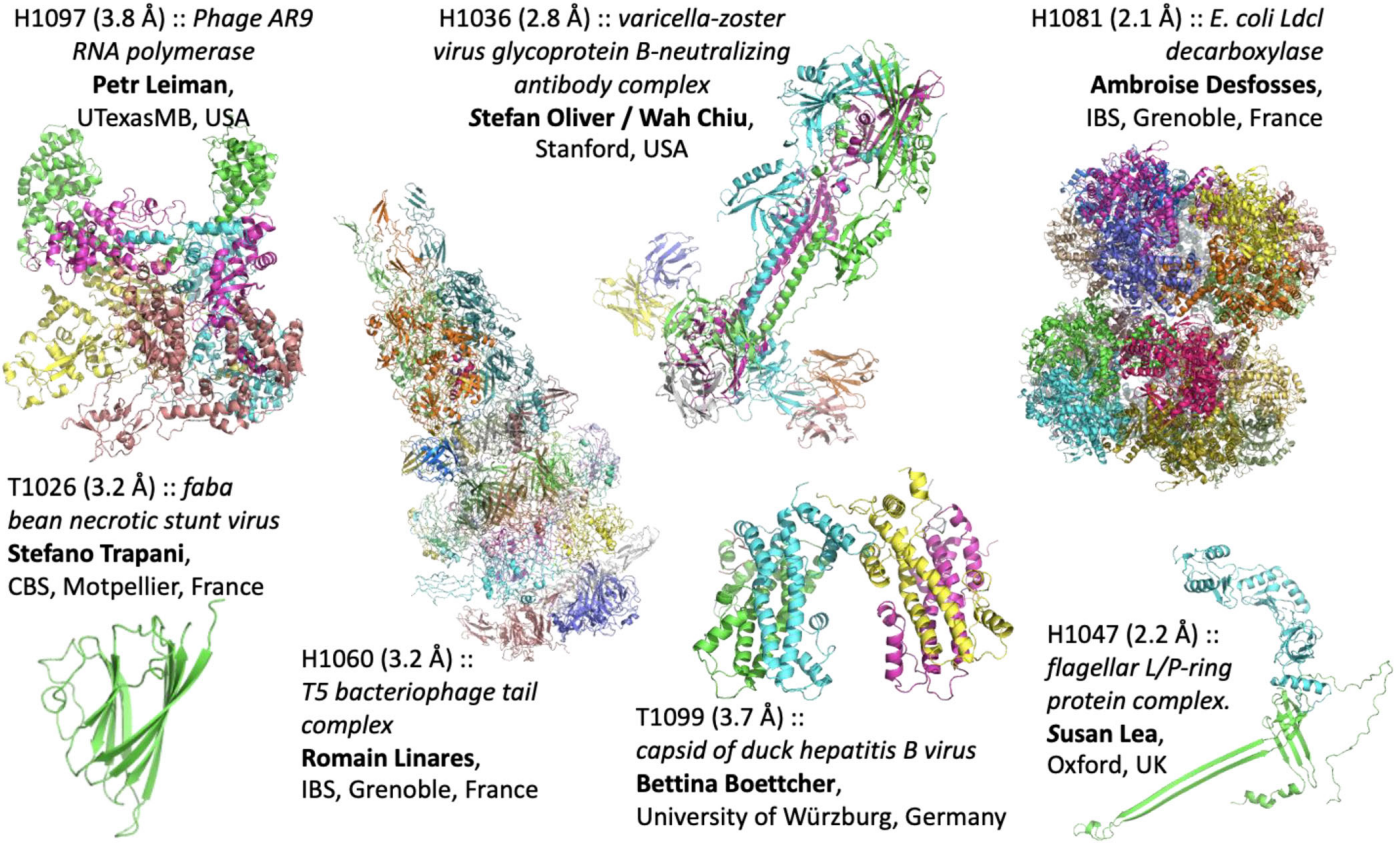
CASP14 cryo‐EM targets. The target code, description, and provider of the reference map and structure are stated next to each target

Seven cryo‐EM groups provided targets for CASP14 (Figure 1). Five of the targets (CASP IDs starting with “H”) were hetero‐multimeric complexes: H1036 (VZV‐gB),^3^ H1047 (L/P‐ring),^4^ H1060 (T5), H1081 (LdcI), H1097 (AR9 RNAP); and the remaining two were homo‐multimers: T1026 (FBNSV) and T1099 (DHBV).^5^ The structures span a resolution range between 2.1 and 3.8 Å, and vary in length from 140 to 949 residues for individual subunits. For comparison, in CASP13 the corresponding numbers were 3.0 to 4.0 Å and 149 to 848 residues, respectively.

As is customary in CASP, for evaluation, targets are split into subunits and domains. With such a procedure, CASP14 cryo‐EM entries yielded 13 single‐domain evaluation units (EUs), four multi‐domain EUs, and six multimeric EUs.

### 2.3 Predictive difficulty of targets

CASP14 evaluation units were assigned to three broad prediction difficulty categories—easy (or TBM), medium (or TBM/FM) and hard (or FM)—based on the template availability and performance of predictors. More details on the principles of assigning targets to different prediction difficulty categories can be learned from other papers of this issue (Kinch et al, Target Classification^6^; Karaca et al, Assessment of Oligomeric targets^7^). Table 1 summarizes information on the difficulty of CASP14 evaluation units emanating from cryo‐EM targets.

**TABLE 1:** Overview of evaluation units from CASP14 cryo‐EM targets

	System	Evaluation unit	Type	Target length	Difficulty	# Models GDT_TS > 70 (monomers) or LDDT > 70 (multimers)
1	FBNSV	T1026	Single‐domain	146	Easy	59
2	VZV‐gB	T1036s1	Single‐domain	621	Easy	77
3	AR9 RNAP	T1092‐D1	Single‐domain	245	Easy	5
4	AR9 RNAP	T1092‐D2	Single‐domain	181	Easy	112
5	AR9 RNAP	T1093‐D1	Single‐domain	141	Hard	22
6	AR9 RNAP	T1093‐D2	Single‐domain	382	Easy	5
7	AR9 RNAP	T1093‐D3	Single‐domain	106	Hard	5
8	AR9 RNAP	T1094‐D1	Single‐domain	277	Easy	5
9	AR9 RNAP	T1094‐D2	Single‐domain	207	Hard	83
10	AR9 RNAP	T1095	Single‐domain	649	Easy	2
11	AR9 RNAP	T1096‐D1	Single‐domain	255	Hard	12
12	AR9 RNAP	T1096‐D2	Single‐domain	171	Hard	27
13	DHBV	T1099	Single‐domain	178	Easy	5
14	AR9 RNAP	T1092	Multiple‐domain	426	Medium	5
15	AR9 RNAP	T1093	Multiple‐domain	631	Hard	0
16	AR9 RNAP	T1094	Multiple‐domain	496	Hard	5
17	AR9 RNAP	T1096	Multiple‐domain	464	Hard	0
18	VZV‐gB	H1036	Complex	856	Medium	22
19	LdcI	H1081	Complex	758	Medium	0
20	AR9 RNAP	H1097	Complex	2682	Hard	0
21	DHBV	T1099ov0	Complex	262	Medium	0
22	L/P‐ring	H1047	Complex	597	Hard	0
23	T5	H1060	Complex	1106	Medium	0

*Note*: Targets with models of acceptable accuracy (LDDT > 70, last column) are marked blue in the Evaluation Unit column; red otherwise. Easy/medium/hard targets are marked green/yellow/orange in the Difficulty column.

### 2.4 Minimum accuracy of models for evaluation

Evaluation of models versus maps makes sense only if models are of high accuracy enabling sensible fitting in the density. Here we define “high accuracy” models as those scoring in excess of 70 LDDT and GDT_TS for monomers, and 70 LDDT for multimers versus the reference experimental structure. This cutoff was selected as a trade‐off between the accuracy of models and the number of targets and models suitable for evaluation.^8^ Table 1 provides the number of models satisfying this criterion for all cryo‐EM evaluation units.

### 2.5 Evaluation measures

The models submitted for each cryo‐EM target were evaluated for their goodness‐of‐fit in the experimental cryo‐EM density map (model‐to‐map goodness‐of‐fit) with nine evaluation measures. The overall goodness‐of‐fit was quantified using TEMPY's 2.0^9^ cross‐correlation coefficient (CCC) and Mutual Information (MI) score^10^, ^11^; PHENIX's^12^ real space correlation coefficients—CCvolume, CCmask, and CCpeaks—each probing different aspects of model‐to‐map fit^13^; and the Atom Inclusion score.^14^ The local (per‐residue) goodness‐of‐fit is evaluated with PHENIX's CCbox measure,^13^ TEMPy's SMOC score,^10^, ^15^ and EMringer score.^16^

The cross‐correlation coefficients are computed between the experimental map with model‐derived maps produced to a specified resolution limit on the same voxel grid, integrated either over the full map or selected masked regions. The TEMPY *CCC* and PHENIX *CCbox* [0;1] coefficients quantify real space cross‐correlation between the entire target map and the map calculated from the model coordinates. The two coefficients are highly correlated, but not identical, owing to slightly different approaches in computing the scores. Both approaches use the entire map for the calculation, but TEMPY directly calculates the product of densities at the same points in the maps, while PHENIX first offsets density values so that the mean of the density distribution is zero, and only then takes the product of the corresponding resulting values. PHENIX *CCvolume* [0;1] expresses correlation between a model and target density map regions with the highest density values. These regions are defined by the N highest value points in the model‐calculated map, with N being the number of grid points inside the molecular mask. PHENIX *CCmask* [0;1] evaluates correlation between a model and target density map values inside a mask calculated around the macromolecules. PHENIX *CCpeaks* [0;1] scores correlation between a model and target density map regions with the highest density values. The regions are defined by the N highest value points in the model‐calculated map and the N highest value points in the experimental map. TEMPY *MI* [0;∞] is a mutual information based score, a statistical measure that compares binned densities relative to their background distributions; larger values correspond to better fits. The EMDB *Atom Inclusion* score [0;1] determines the fraction of atoms inside the map at a specified density threshold. *EMRinger* score [0;∞] evaluates correctness of backbone positioning by measuring the peak positions of unbranched protein C_γ_ atom positions versus map density in ring‐paths around *C*_ɑ_–*C*_β_ bonds. Most carefully refined structures score above 1.5, with some getting scores above 3. The TEMPy Segmented Manders Overlap Coefficient (*SMOC*) score [0;1] represents the Mander's overlap coefficient for overlapping residue fragments: it is computed on local spherical regions around the seven residues in the current window. Overlapping windows are used, producing one numerical value per residue. Local correlation coefficients, SMOC and EMringer scores can be generalized for the whole structure by averaging the per‐residue scores.

The paper also summarizes the results of model evaluation versus cryo‐EM reference structures (that is, models generated by the experimentalists using cryo‐EM map). This analysis serves the purpose of ensuring that there are no irregularities in ranking participating groups on cryo‐EM targets compared to all targets.^29^, ^30^ Evaluation measures and principles for ranking participating groups are described in our CASP13 evaluation paper.^1^ Four measures are used in this type of analysis: a rigid‐body structure superposition measure GDT_TS,^17^, ^18^ and three superposition‐free measures—LDDT,^18^ CADaa,^19^ and SphereGrinder (SG).^20^

CASP infrastructure for running the evaluation, reporting scores and visualizing evaluation results for cryo‐EM targets (http://predictioncenter.org/casp14/cryoem_results.cgi) was designed on the prototype of the evaluation infrastructure^21^, ^22^ developed for the cryo‐EM model challenges.^1^, ^23^

### 2.6 Model refinement in map

We refined an atomic model in the density map by using a Gaussian Mixture Model to represent the protein structure and refine it in the map.^24^ We compute a responsibility map, which is an intensity‐weighted map for each atom, based on their position and the position of all other atoms. This gives us the new expected (mean) position of every given atom, based on the intensity of each voxel in the original map, and the weight of each voxel in the responsibility map:

$$x_{\mathrm{new}}=\left|\frac{G\left(x_{i},\sigma \right)}{\sum_{i}G\left(x_{i},\sigma \right)}⊙M\right|,$$

with *M* being the reference map, *G* a Gaussian function, and *x*_*i*_ denoting the current position of an atom, $x_{i}^{\mathrm{new}}$ the new estimated position, and the average performed over the Cartesian coordinates, weighted by the value computed in the equation. This procedure is repeated several times, alternating with minimization cycles using the Amber ff14sb forcefield,^25^ to maintain correct stereochemistry. This procedure is repeated for five cycles.

### 2.7 Map segmentation

To accurately gauge whether a model is an accurate reflection of the intensity generated by the target of interest, the maps were masked using a procedure where the fitted model was used to scale the voxel intensities, with voxels further from the model scaled lower, depending on their distance to the target model and other models, using a Gaussian distribution for each atom. This resulted in the intensity of voxels closer to other chains that were not targets to be scaled down.

## 3 RESULTS

### 3.1 Evaluation versus reference structure

**FIGURE 2:**
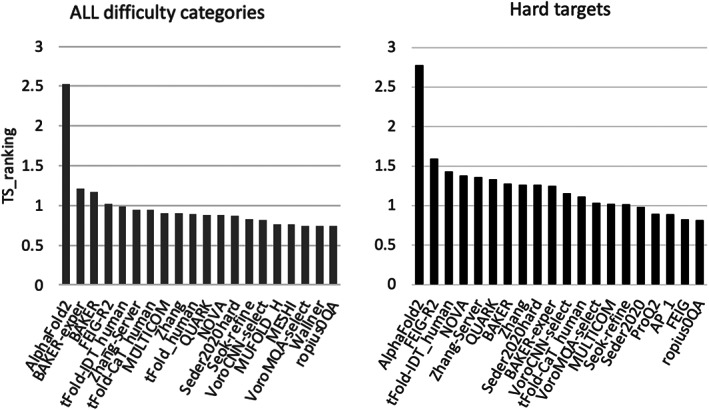
Relative performance of CASP14 participants on cryo‐EM targets in terms of TS_ranking score.^1^ (Y‐axis) Left panel shows ranking on all cryo‐EM targets based on the best model out of five for each group; right on the subset of free modeling (FM) domains

To compare the performance of participants on cryo‐EM targets, we apply the ranking procedure described in our previous evaluation paper.^1^ Figure 2 provides a summary of the relative performance of groups on all cryo‐EM targets (left) and hard cryo‐EM targets only (FM domains, right). Group AlphaFold2 demonstrated outstanding performance in both scenarios, being more than 2.5 SDs above the average scores. These results are in agreement with the results of evaluations on all targets (Figure S1).

### 3.2 Evaluation of model‐to‐map fit

Evaluating the goodness‐of‐fit of CASP models to the experimental cryo‐EM density maps makes sense only for targets where high‐accuracy models are available. Thus, we ran evaluations only on accurate models (see Methods) of the targets marked blue in Table 1. The main aim of this analysis was to check if CASP models, which were built without the knowledge of density maps, could be further refined into the density so that they can reach the quality and goodness‐of‐fit of the models provided by experimentalists (reference structure).

**FIGURE 3:**
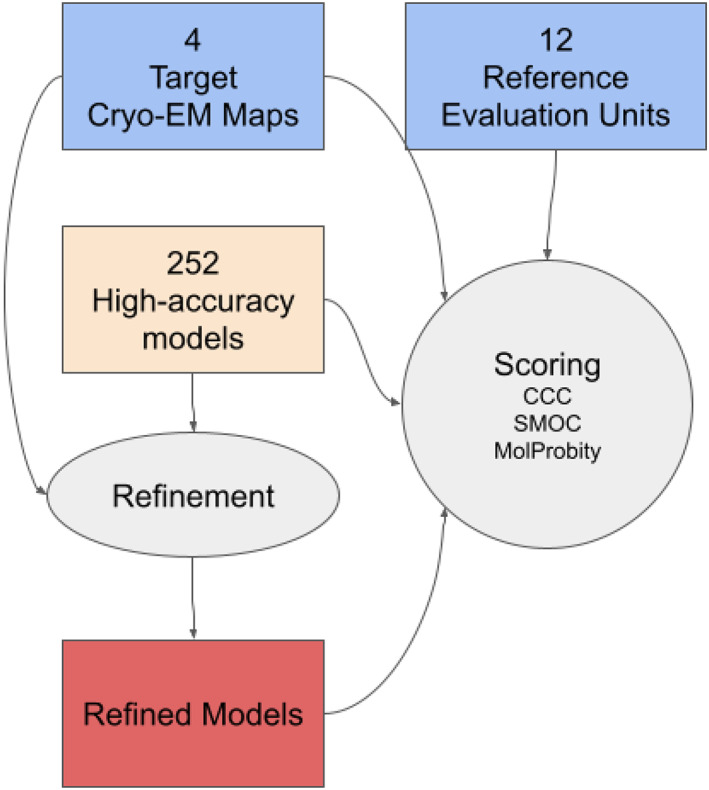
Refinement and assessment pipeline applied to the high‐accuracy CASP models of cryo‐EM targets

To examine this, we applied an automated refinement protocol (Figure 3) to the high‐accuracy models of 12 EUs from four targets, and compared goodness‐of‐fit to the map of the refined models and the reference ones. We used our in‐house real‐space refinement implementation in TEMPy (with openMM^26^) with AMBER14^25^ forcefield and five macro‐cycles (see Section 2). We then assessed the refined models and compared them to the original models.

We compared all the scores (prior to refinement), in order to understand the relation between them, by computing all‐against‐all correlation matrices, across all targets. Unsurprisingly, most scores exhibit a high degree of correlation (calculated only on targets with more than 10 high‐accuracy models), with the exception of EMringer scores, as seen previously^1^ (Figure S2).

**FIGURE 4:**
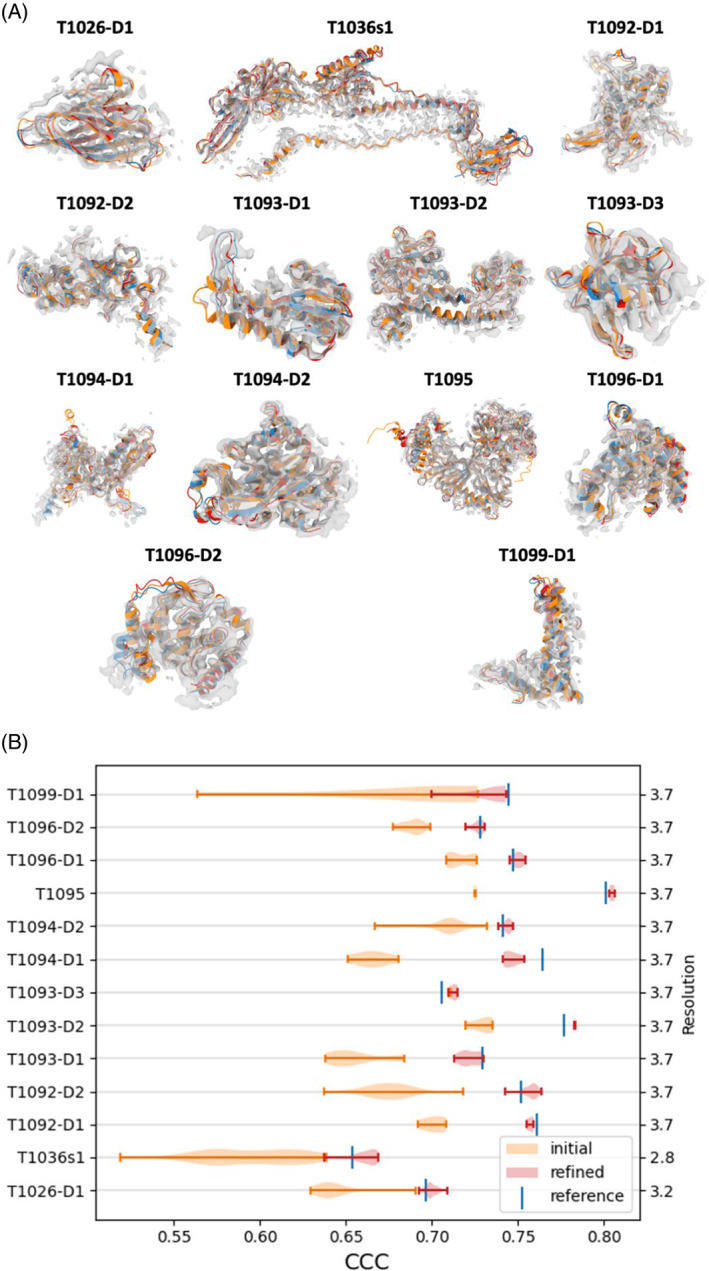
(A) Single domain refinement: Reference structure (blue), best model (orange), best model after refinement (red), against the map, contoured to only show high‐intensity voxels (gray). Bright colors indicate that the structure is outside the density. (B) Average CCC (TEMPy) before and after refinements, across targets, compared to the reference structure. The refinements significantly improve the average CCC for all targets. Target name is on the left axis and resolution is on the right

Following refinement, the global improvement of the models relative to their corresponding reference structure is shown in Figure 4. The overall quality‐of‐fit to the map has improved significantly (Student's *t*‐test with a statistic of 21.88 and a *p* value of 5.00e‐71) indicating that in general, CASP models can be further improved in the presence of the cryo‐EM maps (even when the initial models are of very high quality). This improvement can be attained without sacrificing geometrical fidelity of models as the average MolProbity^27^ score of the refined models remained similar (with an average MolProbity score change of 1.93).

### 3.3 Local improvement of models compared to reference structure

**FIGURE 5:**
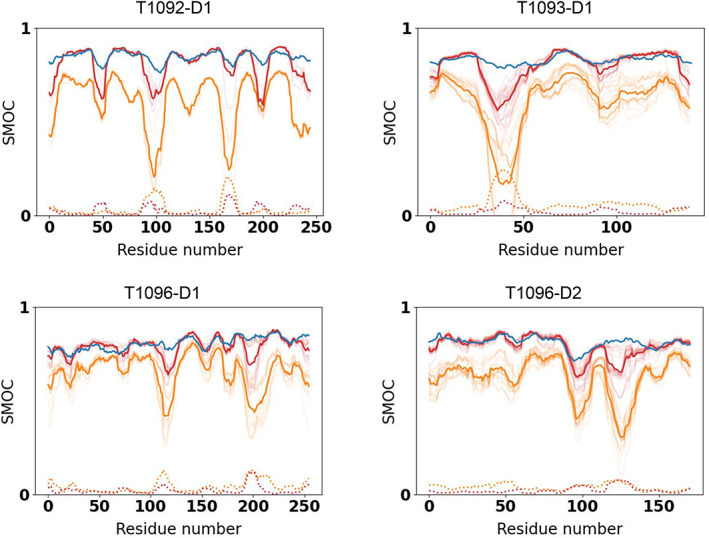
SMOC score for the reference structure (blue), high‐accuracy CASP models (orange) and the same models after refinement (red) for three selected targets. The transparent lines represent SMOC for individual models and the average of the SMOC scores is shown in a thick line while SD is shown in a dotted line before (orange) and after (red) refinement

To quantify the differences in goodness‐of‐fit of CASP models (refined and unrefined) versus the reference structure we calculated the Pearson correlation coefficient between the residue‐dependent SD of their SMOC scores. We found that the SD is anticorrelated with the reference SMOC score, while the mean of the SMOC in the refined models is correlated with the reference SMOC score (mean Pearson correlation coefficient across all targets is −0.44 and 0.76, respectively). This result indicates that regions of lower quality‐of‐fit in the reference structure tend to exhibit higher variability among CASP models (prior to refinement). Figure 5 illustrates improvement in coordinates‐to‐map fit for four selected targets (plots for all nine remaining targets are shown in Figure S3). The graphs show that the fit improved substantially during the refinement, reaching the accuracy of the reference in many cases. The regions with high SD tend to be less refinable.

### 3.4 Local improvement of specific elements

To understand the improvement in CASP models following density‐based refinement, we examined specific cases where such models resulted in structural elements that are fitted equally well or better than the reference in the cryo‐EM map. We provide three examples, representing loops, secondary structural shifts and domain orientation.

#### 3.4.1 Loops: AR9 RNA polymerase (T1092‐D1)

**FIGURE 6:**
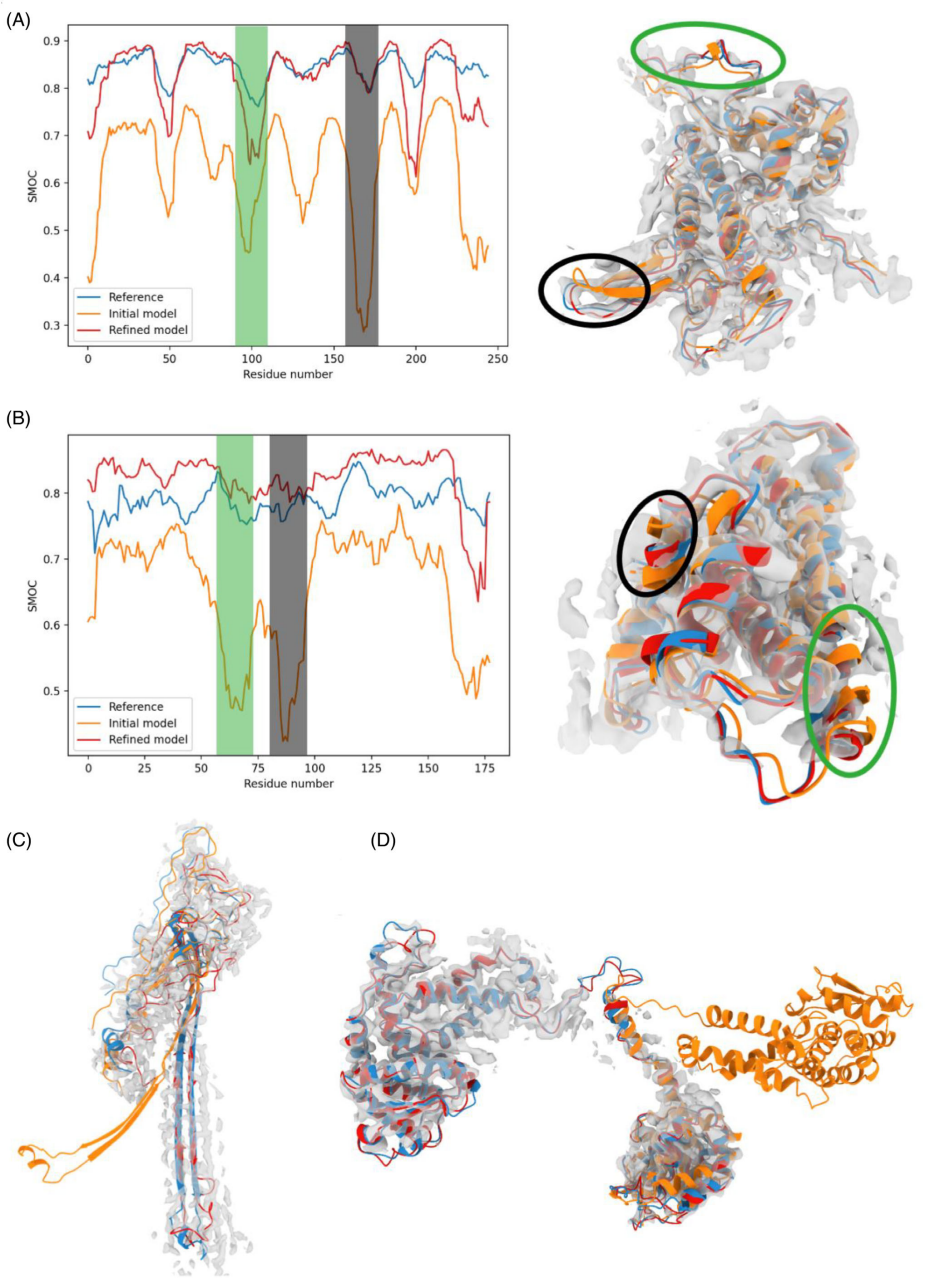
Examples of improvement in structural elements in the refined CASP models: (A) (T1092‐D1) and (B) (T1099). Left: SMOC scores for the reference structure (blue), best predicted structure before refinement (orange) and after (red). The gray and green shaded areas indicate regions of lower score in the predicted structure, highlighting a locally worse fit. Right: Representation of the structures within the map. The regions surrounded by a blue and black oval correspond to the shaded areas in the left diagram. (C) Structure of the reference L/P flagellar ring structure subunit T1047 (blue), best predicted model (orange) and after refinement (red). (D) Structures of the reference structure T1096 (blue), and best predicted model before refinement (orange) in the map

In this example, most of the structure of the top‐predicted model (AlphaFold2) is well fitted, achieving a local SMOC score of around 0.7, with only a few loops outside the density (Figure 6A). Following our refinement protocol, most of the model achieved a quality similar to the reference structure.

#### 3.4.2 SSE shifts and rotation: duck virion hepatitis B (T1099) and flagellar L/P ring (T1047)

In the predicted model for T1099 (by AlphaFold2), the overall topology of the structure is correctly predicted, but there is a shift in the position of two α‐helices with respect to the reference structures (RMSD of ~2 Å for the those regions) (Figure 6B, highlighted in green and black circle (left) and shaded areas (right)). The refinement procedure pushed these helices into the density, resulting in a model with an overall quality slightly higher than the reference (average SMOC score of 0.83 for the refined model and 0.79 for the reference structure).

The predicted mode for T1047 (Figure 6C) is another example of SSE movement. The top predicted model for this target (AlphaFold2) is not in the original list of structures we selected for refinement due to its low GDT_TS (50.4) (although LDDT was above the cutoff—75). However, in this case, the fold is partly correct with the SSEs slightly rotated and therefore we decided to test if the accuracy of the structure can be improved with refinement.

#### 3.4.3 Domain orientation: AR9 RNA polymerase (T1096‐D1‐D2)

In this example, both domains of the T1096 subunit of the RNA polymerase were correctly predicted by AlphaFold2 (with very high accuracy: GDT‐TS of 83.63 and 78.80) (Figure 6C). However, the linker between the two domains was predicted incorrectly, resulting in a wrong orientation between them. In this case, refining the model in the density map easily fixed the problem.

## 4 DISCUSSION

A sizeable portion of CASP14 targets (22% of EUs) was determined with cryo‐EM. The accuracy of the submitted models for cryo‐EM targets is equivalent to that for X‐ray targets. Not surprisingly, the ranking of the participating groups on cryo‐EM targets is consistent with those on all CASP14 targets, with the AlphaFold2 group topping the rankings, with a big lead over other participants. As cryo‐EM structures tend to differ from X‐ray or NMR structures in their size or complexity of quaternary structure, it is interesting to look at predictions at the complex assembly level. Unfortunately, despite the high accuracy of the individual protein level predictions, the prediction of quaternary structures was not successful, possibly due to the size and complexity of those assemblies.^7^

Refinement of the submitted CASP models in the experimental density shows that the models could be improved to the point of approaching the quality of the reference structures (and beyond in some structural elements), thus indicating that high‐quality models from CASP predictors can be a good starting point for structure refinement. These structures often represent large complexes, where many proteins have to be predicted, some of which can only be modeled accurately in the context of other proteins. Starting refinement in the experimental map using CASP models can be useful even when there are domain orientation differences (as seen in T1096) or SSE shifts and rotation (as seen in T1047 and T1099), as these errors can easily be fixed. This could potentially save computer time and reduce the overall effort in reaching a good model, prior to manual adjustments with tools (such as *Coot* ^28^), especially for loop regions that are more ambiguous. An example of practical application of modeling to cryo‐EM structure determination is described in another paper of this issue,^31^ which discusses how the AR9 polymerase experimental model was built with the guidance of CASP models.

The anticorrelation observed between the SD of the SMOC scores in the unrefined models and the reference structures is likely due to the intrinsic dynamic property of some regions, that is captured to some extent by both the cryo‐EM experiment, and the ensemble of prediction models represented in CASP. These regions may exhibit higher flexibility (from either disorder or alternative conformers), resulting in locally lower resolution in the map, leaving the density in the region poorly resolved; this would further explain the difficulty in refining those regions, and maybe suggest that it is better to describe these regions with multiple conformers rather than one.^10^ Potentially CASP models could be used to estimate zones of increased difficulty, both experimentally and computationally, by looking at the local divergence in an ensemble of structures generated by different prediction methods.

The work presented here shows that sequence‐based prediction with subsequent refinement can now rival the quality of reference models. The correlation between the reference structure quality and the variability in predicted structures provides a new avenue to identify regions of uncertainty in modeling approaches. We see cryo‐EM structures becoming an important player in future CASP experiments, potentially helping the development of better prediction methods for protein dynamics and assembly.

### PEER REVIEW

The peer review history for this article is available at https://publons.com/publon/10.1002/prot.26216.

## Supporting information


**APPENDIX S1**: Supporting information

## Data Availability

Data sharing is not applicable to this article as no new data were created or analyzed in this study.
