# Supplementary material for: Cryo-EM targets in CASP14
Source: Proteins. Author manuscript; Available in PMC 2022 Dec 1. (PMC8630773; doi:10.1002/prot.26216)
Supplement: Supplementary file 1 [file NIHMS1733788-supplement-supinfo.docx]

# Supplementary

| Target | Model number | Before | After | Reference |
| --- | --- | --- | --- | --- |
| T1026 | 427 1 | 0.77 | 0.87 | 0.86 |
| T1036 | 487 1 | 0.38 | 0.65 | 0.82 |
| T1092 | 427 1 | 0.72 | 0.91 | 0.89 |
| T1099 | 427 1 | 0.76 | 0.89 | 0.85 |

**Supplementary Table 1.** TEMPy CCC change between best model, before and after refinement, compared to the reference structure

**Supplementary Figure S1.** Relative performance of CASP14 participants on targets determined by all structure determination methods in terms of TS_ranking score[^1^](https://paperpile.com/c/2C0toZ/VreDz) (Y-axis). Left panel shows ranking on all targets based on the best model out of 5 for each group; right on the subset of free modelling (FM) domains.

| T0126-D1 | T1036s1 | T1092-D2 |
| --- | --- | --- |
|  |  |  |
| T1094-D2 | T1096-D1 | T1099-D1 |
|  |  |  |

**Supplementary Figure S2.** Correlation matrices of all the scores, for all targets. The scores were computed on each target independently as the linear relationships between scores appear not to be preserved across targets.

| T0126-D1 | T1036s1 | T1092-D2 |
| --- | --- | --- |
|  |  |  |
| T1093-D2 | T1093-D3 | T1094-D1 |
|  |  |  |
| T1094-D2 | T1095 | T1099-D1 |
|  |  |  |

**Supplementary Figure S3.** SMOC score for the reference structure (blue), high-accuracy CASP models (orange) and the same models after refinement (red) for the targets not shown in Figure 5. The transparent lines represent SMOC for individual models and the average of the SMOC scores is shown in a thick line while standard deviation is shown in a dotted line before (orange) and after (red) refinement. 

| Target ID | Pearson (mean) | Pearson (std) | Spearman (mean) | Spearman (std) |
| --- | --- | --- | --- | --- |
| T1026-D1 | 0.603 | -0.491 | 0.693 | -0.471 |
| T1036s1 | 0.043 | 0.261 | 0.055 | 0.255 |
| T1092-D1 | 0.864 | -0.526 | 0.900 | -0.606 |
| T1092-D2 | -0.056 | 0.259 | -0.057 | 0.182 |
| T1093-D1 | 0.676 | -0.500 | 0.630 | -0.235 |
| T1093-D2 | 0.738 | -0.261 | 0.676 | -0.224 |
| T1093-D3 | 0.707 | 0.0630 | 0.703 | 0.101 |
| T1094-D1 | 0.438 | -0.292 | 0.306 | -0.360 |
| T1094-D2 | 0.858 | -0.559 | 0.885 | -0.505 |
| T1096-D1 | 0.579 | 0.055 | 0.617 | -0.237 |
| T1096-D2 | 0.668 | -0.308 | 0.667 | -0.438 |
| T1099-D1 | 0.423 | -0.249 | 0.482 | -0.274 |
| Average | 0.723 | -0.437 | 0.763 | -0.444 |

**Supplementary Table 2.** Correlations between the SMOC scores of the reference against the mean and standard deviation of the predicted models, using Spearman and Pearson correlation.
